# The impact of economic factors on migration considerations among Icelandic specialist doctors: a cross-sectional study

**DOI:** 10.1186/1472-6963-13-524

**Published:** 2013-12-18

**Authors:** Ingunn Bjarnadóttir Solberg, Kristinn Tómasson, Olaf Aasland, Reidar Tyssen

**Affiliations:** 1 Department of Behavioural Sciences in Medicine, Institute of Basic Medical Sciences, Faculty of Medicine, University of Oslo, P.O.B. 1111 Blindern, 0317 Oslo, Norway; 2 Department of Research and Occupational Health, Administration for Occupational Health and Safety, 110 Reykjavík, Iceland; 3 Institute for Studies of the Medical Profession, The Norwegian Medical Association, P.O.B. 1152 Sentrum, N-0107 Oslo, Norway; 4 Department of Health Management and Health Economics, Institute of Health and Society, P.O.B. 1089, 0318 Oslo, Norway

**Keywords:** Doctors’ migration, Health-care services, Economic recession, Cost-containment initiatives, Doctors’ job satisfaction, Iceland

## Abstract

**Background:**

Globalization has facilitated the employability of doctors almost anywhere in the world. In recent years, the migration of doctors seems to have increased. However, we lack studies on doctors’ migration from developed countries. Because the economic recession experienced by many countries might have affected the migration of doctors, research on this topic is important for the retention of doctors. Iceland was hit hard by the economic recession in 2008. Therefore, we want to explore how many specialist doctors in Iceland have considered migrating and whether economic factors at work and in private life, such as extensive cost-containment initiatives at work and worries about personal finances, are related to doctors’ migration considerations.

**Methods:**

In 2010, all doctors in Iceland registered with the Icelandic Medical Association were sent an electronic cross-sectional survey by email. The 467 specialists who participated in this study represent 55% of all specialist doctors working in Iceland. Information on doctors’ contemplation of migration was available from responses to the question: “Have you considered moving and working abroad?” The predictor variables in our logistic regression model are perceived cost-containment initiatives at work, stress related to personal finances, experience of working abroad during vacations, job dissatisfaction, job position, age, and gender.

**Results:**

Sixty-three per cent of Iceland’s specialist doctors had considered relocation abroad, 4% were moving in the next year or two, and 33% had not considered relocating. Logistic regression analysis shows that, controlling for age, gender, job position, job satisfaction, and experience of working abroad during vacations, doctors’ migration considerations were significantly affected by their experiences of cost-containment initiatives at work (odds ratio (OR) = 2.0, *p* < 0.01) and being stressed about personal finances (OR = 1.6, *p* < 0.001). Age, job satisfaction, and working abroad during vacations also had an effect, whereas job position did not.

**Conclusions:**

Economic factors affect whether specialist doctors in Iceland consider migration. More studies on the effect of economic recession on migration by doctors are needed.

## Background

The well-documented migration of health-care professionals from developing countries may threaten the viability of health-care systems in many countries [1]. However, there is limited evidence on the migratory movement of health professionals from developed countries. Although many countries were affected by the worldwide recession in 2008 [2], Iceland was one of the first countries in Europe to be severely affected by the recession (October 2008) [2-5]. According to the United Nations Development Programme [6], Iceland was the best country in the world to live in before the economic crisis. Its health policy performance is known to be one of the best in Europe [7]. Iceland’s health care is financed by general taxation and governmental reimbursement.

Despite its small population of 320,000, Iceland’s doctor density is relatively high (one per 272 citizens) [8]. However, having only a small population, Iceland has only a small number of specialists in each specialty and so is particularly vulnerable to emigration by specialists.

After the economic crisis, from 2008–2010, health expenditure in Iceland decreased by 5% per year as a result of general expenditure cuts [9]. The total expenditure of the Landspitali University hospital (the main hospital in Iceland) increased by 1% between 2008 and 2010 [10,11]. Over the same period, the index of imported products increased by 48% [8]. In this period, the hospital reduced its costs by 16% by reducing both paid overtime and the number of doctors and nurses on call, limiting diagnostic tests, economizing on pharmaceutical costs, and outsourcing support functions, among other things [9]. In addition, doctors’ salaries increased by only 3.7% between 2008 and 2009 [12], during which time the Consumer Price Index (CPI) increased by 27% [8].

It is not known whether recessions and related factors influence doctors’ contemplation of moving and working abroad. Such an influence may threaten the current structure of Iceland’s health services. Because specialists’ medical training, except in psychiatry and general practice/family medicine, are not available in Iceland, most specialist doctors working in Iceland have experienced temporary migration. During the last 3 years, Iceland’s net migration has been negative, with 8,373 more people emigrating than immigrating. (On 1 January 2012, the total population was 319,575 [8]). Before the crisis, between 2004 and 2008, net migration was positive (15,921 persons) [13].

Although job dissatisfaction has been linked to migration and retirement among doctors [14,15], it remains to be seen whether extensive cost-containment initiatives at work are also related to migration among Icelandic doctors, when adjusted for other factors. Many doctors who have undertaken specialist training abroad return to Iceland in considerable debt and many take up additional loans in order to settle down. It is therefore likely that the economic crisis affected doctors’ private lives as well and concerns about personal finances may have influenced their decision to migrate. Some Icelandic doctors work abroad during vacations, which is often where they became specialists [16]. It is not known how many specialists work abroad during vacations, but it is likely to have been increasing during the recession given that doctors get paid two or three times more in countries such as Norway and Sweden [16]. Working abroad during vacations may prompt specialists to migrate. Job position might also have an effect by influencing job satisfaction and thus migration consideration [17,18]. In addition, the proportion of female specialist doctors is increasing, which may be important. Marital status and place of residence (rural or urban) may also be important determinants of doctors’ migration decisions [14]. Because the economic recession affects other European countries, studying its effect on the availability and supply of specialists may be of interest beyond Iceland.

Given this background, we hypothesize that, controlling for other background and work-related factors, extensive cost-containment initiatives at work and worries about private finances might prompt Icelandic doctors to move abroad.

## Methods

### Participants, data collection, and data analysis

All doctors in Iceland who had registered their email address with the Icelandic Medical Association (96% of all working doctors, n = 1024) were sent our Internet-based electronic survey in March 2010, with four follow-up reminders. The study was reported to the Data Protection Authority (Persónuvernd) and approved by the National Bioethics Committee (Vísindasiðanefnd). The response rate was 61% (622/1024). We used only data on doctors who had completed their specialization (75% of all respondents, n = 467), who constitute 55% (467/846) of all specialists working in the country in March 2010. We sampled only specialist doctors because most non-specialist doctors must migrate to become specialists. The majority (82%) of the specialist doctors lived in Reykjavik at the time of the study. Women made up 30% of our sample, although only 25% of the specialists working in the country in March 2010 were female. Table 1 reports the specialties of all working doctors in Iceland in March 2010 and the specialists in our study. There was no significant difference in the number of doctors in each speciality.

**Table 1:** Areas of specialization of all specialists in Iceland and of specialists in our study, March 2010

**Specialty**	**Our sample**	**All specialists in Iceland**
General practice/family medicine	100 (23.9%)	173 (20.4%)
Internal medicine	161 (38.4%)	308 (36.4%)
Surgery/gynaecology	91 (21.7%)	224 (26.5%)
Psychiatry	32 (7.6%)	68 (8.0%)
Laboratory, community medicine, administration, and other stated specialty	35 (8.3% )	73 (8.7%)
Sub-total*	419 (100%)	846 (100%)
Total specialists, including those with specialty not indicated	467	846

*48 specialists did not indicate their specialty, but only noted the type of facility (such as hospital or general practice).

### Dependent variable

#### Consideration of moving abroad

We asked the question: “Have you considered moving and working abroad?” Precoded responses were: 1) No; 2) Yes, I have considered it, but for various reasons, it is not possible; 3) Yes, I have considered it; and 4) Yes, I am moving abroad in the next 2 years. To undertake logistic regression, we coded response 1) as 0 (those who had not considered moving) and responses 2), 3), and 4) as 1 (those who had considered moving). For validation, we also used responses 1) and 2) to represent non-movers and responses 3) and 4) to represent potential emigrants.

### Independent variables

Age was categorized into nine levels: 1) 29 years and younger; 2) 30–34 years; 3) 35–39 years; 4) 40–44 years; 5) 45–49 years; 6) 50–54 years; 7) 55–59 years; 8) 60–64 years; and 9) 65 years or older.

### Job position

To control for the potential effect of job position, the specialist doctors were categorized into hospital doctors, general practitioners (in the Nordic countries, general practice/family medicine is a medical specialty), private practice specialists, and others (specialist doctors who worked in health authorities, specialized institutions, universities, etc.). Given these categories, the reference group is private practice specialists (e.g., “semi-private” specialist doctors who receive part payment directly from the Icelandic Health Insurance and part payment from the patient). These categories have also been used in recent Norwegian studies [17,18].

### The influence of cost-containment initiatives at work

We asked doctors to respond to the following statement: “I find that cost-containment initiatives (such as limited selection of devices and equipment) influence my work” (see Table 2). The five response categories were recoded into two groups: i.e., strongly disagree, disagree, and neither agree nor disagree were coded 0, and agree and strongly agree were coded 1.

**Table 2:** Description of independent variables (n = 467)

**Predictor variables**	**Mean (SD) and %**
Age	6.0* (1.6)
Female	30% (n = 140)
Work position (n = 457)	
Hospital specialist doctors	58.6% (n = 268)
General practitioners/family medicine	21.4% (n = 98)
Private practice specialist doctors	12.3% (n = 56)
Other positions	7.7% (n = 35)
Job satisfaction (n = 444)	
Mean (SD)	48.5 (11.4)
Median	49.0
Cost-containment initiatives’ influence on work (n = 456)	
1. Strongly agree	33.3% (n = 152)
2. Agree	41.7% (n = 190)
3. Neither agree nor disagree	14.5% (n = 66)
4. Disagree	8.1% (n = 37)
5. Strongly disagree	2.4% (n = 11)
Worries about personal finances (n = 462)	
1. Not stressful	36.6% (n = 169)
2. Slightly stressful	31.0% (n = 143)
3. Stressful	18.6% (n = 86)
4. Quite stressful	9.5% (n = 44)
5. Very stressful	4.3% (n = 20)
Working abroad during vacations	10% (n = 48)

*Group 6 = 50–54 years old. In each group: 29 years and younger = 0.4%; 30–34 years = 0.4%; 35–39 years = 4.1%; 40–44 years = 16.3%; 45–49 years = 19.1%; 50–54 years = 18.9%; 55–59 years = 19.6%; 60–64 years = 16.6%; 65 or older = 4.5%.

### Job satisfaction

Job satisfaction was measured by using the 10-item Job Satisfaction Scale [19]. This measure has previously been used by doctors in Norway and other countries [17,18,20,21]. The variable was dichotomized: those recording at least a median level of satisfaction were coded 0, and those recording below the median level of job satisfaction were coded 1 (dissatisfied).

### Worries about personal finances

We asked the question: “How stressful are worries about personal finances for you?” Respondents chose between: 1) not stressful; 2) slightly stressful; 3) stressful; 4) quite stressful; and 5) very stressful.

### Working abroad during vacations

In answer to our question “Do you work for other employers in vacations?”, respondents chose between: 1) No; 2) Yes, in Iceland; 3) Yes, abroad. The last of these was coded 1, and the other two were coded 0.

The independent variables are described in Table 2.

### Statistics

We performed Student’s *t*-tests for differences in means. Logistic regression analysis was used to estimate the effects of the predictors on moving abroad. We chose a significance level of 5%, and used 95% confidence intervals (CIs). PASW Statistics 20 was used for the statistical analysis.

## Results

Forty-seven per cent of the specialist doctors (n = 218) had considered moving; 16% (n = 73) had considered moving but, for unspecified reasons, had been unable to; 4% (n = 16) said they were moving in the next year or two; and 33% (n = 155) had not considered moving abroad. The doctors who had considered moving (including those who were unable to move), or were soon to move (67% in total) were less satisfied in their job than were doctors who had not considered moving: the respective means for job satisfaction for these two groups are 45.3 (with a standard deviation (SD) of 10.8) and 51.8 (SD = 11.0). Testing for a difference in these means yields t = -6.3 (with *p* < 0.001). Doctors expressing a desire to move also experienced greater cost containment at work (mean = 1.8, SD = 0.9) than those that did not (mean = 2.3, SD = 1.1), t = -4.9 (*p* < 0.001). Doctors wanting to move were more stressed about personal finances (mean = 2.4, SD = 1.2) than those who were not (mean = 1.8, SD = 0.9), t = 5.6 (*p* < 0.001). Ten per cent of doctors had worked abroad during vacations (n = 48) and 90% of these had considered moving abroad or were about to do so. The adjusted predictors of considering moving abroad are reported in Table 3. We found that type of specialty, whether working outside Reykjavik, and having a partner (represented by marital status) were not significant predictors. According to our adjusted analysis, the significant determinants of doctor emigration are being young (OR = 0.7, *p* < 0.001), experiencing job dissatisfaction (OR = 2.9, *p* < 0.001), having experience of cost-containment initiatives (OR = 2.0, *p* < 0.01), being stressed about personal finances (OR = 1.6, *p* < 0.001), and having worked abroad during vacations (OR = 5.5, *p* < 0.01).

**Table 3:** Predictors of doctor considerations of moving abroad (considering/moving = 67%, not considering moving = 33%, n = 430 in the adjusted analysis)

	**Unadjusted**		**Adjusted**	
Covariates	OR	95% CI	OR	95% CI
Age	0.63***	0.55 to 0.72	0.71***	0.60 to 0.84
Male	0.89	0.58 to 1.37	1.50	0.85 to 2.52
Private practice (ref)				
General practice/family medicine	1.06	0.66 to 1.70	1.01	0.44 to 2.32
Other	0.55	0.27 to 1.11	1.23	0.44 to 3.43
Hospital	1.41	0.95 to 2.09	1.39	0.68 to 2.84
Job dissatisfaction	4.74***	3.04 to 7.39	2.88***	1.74 to 4.75
Cost-containment initiatives	2.65***	1.72 to 4.11	2.03**	1.19 to 3.45
Worries about personal finances	2.14***	1.70 to 2.68	1.64***	1.26 to 2.13
Working abroad during vacations	4.93***	1.91 to 12.72	5.50**	1.50 to 20.12

*p < 0.05, **p < 0.01, ***p < 0.001.

We validated this predictor model by using a regression based on only those doctors who could move or were moving and obtained similar results: being younger (OR = 0.8, *p* < 0.001); job dissatisfaction (OR = 2.3, *p* < 0.001); cost-containment initiatives (OR = 2.1, *p* < 0.01); stress about personal finances (OR = 1.2, *p* = 0.09); having worked abroad during vacations (OR = 5.9, *p* < 0.001).

We tested all the significant variables for interaction with gender and found no significant interactions.

## Discussion

Two-thirds of Iceland’s specialist doctors had considered moving abroad and 51% had considered moving and were able to do so. Experiencing extensive cost-containment initiatives, being stressed about personal finances, experiencing job dissatisfaction, having worked abroad during vacations, and being young all had significant effects on doctors’ desires to emigrate, even when controlling for job position. These factors were equally important for both male and female doctors; there were no significant gender interactions.

The migration of specialist doctors has never been an issue in Iceland. When young doctors return home after completing their specialist training in other countries, they usually stay. The high proportion of specialists who considered migrating in our study is therefore unusual and surprising. Some authors have reported that intentions data are only imperfectly correlated with future behaviour [22,23]. On the other hand, studies have confirmed that migration intentions are indeed predictable of subsequent move [24,25].

Moreover, there is evidence that these considerations were acted upon: unpublished data from the Icelandic Medical Association shows that between 2005 to 2012, there was a significant increase in the emigration of Icelandic doctors. Mullans’ emigration factor was 23 in 2005 (318 emigrants and 1060 doctors working in Iceland) and 39 in 2012 (661 emigrants and 1049 doctors working in Iceland), (*p* < 0.0001) [26]. This also reflects the concerns in the Icelandic society that young specialists with postgraduate experience from abroad are not returning to Iceland [27,28]. Since May 2009, the Icelandic Medical Association has kept data on doctors moving to and from Iceland. These are the most accurate data available, since 96% of the Icelandic doctors are registered there. Between May 2009 and February 2013 244 doctors moved from Iceland and only 91 doctors moved back. In a two-year period from May 2009 to April 2011, there were only 30 doctors who returned to Iceland, whereas in the following 22 months 61 doctors returned (personal communication, manager Sólveig Jóhannsdóttir, Icelandic medical association). Actually, this suggests an increase in the number of doctors returning after specialization. Unfortunately, we could not include the doctors abroad in our study. A study of their opinions and intentions to migrate back to Iceland is needed.

We found that specialist doctors who had experienced cost-containment initiatives at work were more likely to consider moving abroad. Seventy-five per cent of the doctors in our study agreed or strongly agreed with the notion that cost-containment initiatives had interfered with their work (see Table 2). As mentioned previously, health expenditure in Iceland decreased following the economic crisis and doctors have had to deal with limits on the affordable number of diagnostic tests, reduced numbers of doctors and nurses on call, etc. [9]. With regard to migration, studies have shown that poor working conditions and a desire for greater access to enhanced technology, equipment, and health facilities can be important [29,30]. To our knowledge, no studies have shown a connection between cost-containment initiatives at work and migration. This is probably because countries with high emigration rates tend to have stable but depressed economies. In Europe, over recent decades, there has been emigration from less wealthy and more turbulent regions to richer ones [31-35]. In Germany, however, the emigration of German doctors is increasing and this is related to doctors’ dissatisfaction with their salaries, among other things [36].

Costigliola’s recent review of the mobility of medical doctors in cross-border health care concluded that one of the most-cited factors for physicians’ mobility is financial motivation [32]. This is consistent with our results: doctors who were more stressed about personal finances were more likely to have considered or decided to move abroad. In our study, however, such worries may be a reflection of the general economic recession in the country, which affects all groups in society. In the Nordic countries, which have democratic socio-political systems and relatively high levels of equity, medical professionals do not earn particularly high wages. As already mentioned, doctors’ salaries increased by only 3.7% between 2008 and 2009 [12], when the CPI increased by 27% [8]. According to Voydanoff, economic stress refers to aspects of economic life that are potential stressors for employees and their families and consists of both objective and subjective components regarding employment and income [37]. The three aspects of economic stress that have been researched thoroughly are unemployment, underemployment, and job insecurity [37]. Although these aspects are not especially relevant to the doctors in our study, the subjective economic stressor (economic strain) is germane. Voydanoff describes economic strain as perceived financial adequacy, financial concerns and worries, and adjustment to change in financial status [37]. Only 37% of the specialist doctors in our study were not worried about personal finances (see Table 2). Well-being and behavioural outcomes are more closely related to subjective economic stress than to objective stressors, and economic stress is known to negatively influence well-being [38]. Our indicator of economic strain, namely doctors’ worries about their private finances, was a significant predictor of whether doctors wanted to migrate, controlling for job satisfaction. Evidence in several studies of an association between doctors’ and nurses’ incomes and their motivation to migrate validates our findings [30,32,39-41]. However, our findings are also unique in that they refer to a stable and previously wealthy European country.

Our results indicate that job dissatisfaction among Icelandic doctors may motivate them to emigrate. With the exception of study of Canadian family doctors [14], to our knowledge, the present study is the first to present evidence of a relationship between migration considerations and job satisfaction in an adjusted model, covering doctors of all specialities. Job satisfaction among Iceland’s doctors requires further study, because it is not only important in relation to migration but is also related to burnout [42], turnover [15,43], and health-care service quality [44]. We found no evidence that job position affected emigration considerations. This emphasizes the fact that such considerations are quite widespread and generalized in the medical profession.

The president of the Icelandic Medical Association, Þorbjörn Jónsson, reports that commuting has increased among doctors after the economic crisis [16]. In our study, ten percent of the specialists had worked abroad during vacations. Because of Iceland’s small population, commuting might help doctors to maintain their skills and increase their experience. Nevertheless, according to our study, 90% of the specialists who had worked abroad during vacations had considered moving or were moving abroad and were five times more likely to have considered migration than those who had not worked abroad during vacations. The doctors get much higher pay abroad [16], which might partly explain these findings.

Even more worrying is the finding in our study that younger specialists are more likely to consider migration than their older colleagues, which confirms a finding among Canadian physicians [14,45]. Because young doctors represent the potential future health-care services of a country, their perceptions about moving abroad have important implications for maintaining a high-quality health-care service in the years to come [46].

A review of physicians’ intentions to withdraw from practice by Williams concluded that the combination of job stress and dissatisfaction can be so powerful that some highly trained and committed professionals may quit while others cope by reducing their work hours, changing practice emphasis, or abandoning direct patient care [47]. A shortage of physicians may impose excessive burdens on many, who then quit, and this may spark a downward spiral that exacerbates the initial shortage, which in turn might lead to even more stress and heavier workloads for the remaining doctors [29]. When doctors migrate, they are likely to form networks overseas that may promote further emigration by colleagues with whom they keep in touch and to whom they might advertise migration opportunities and provide assistance. Evidence of the influence of social networks on migration by nurses is apparent in other studies [48]. Our findings call for appropriate strategies on the part of relevant authorities, focusing on the doctors’ working conditions, such as their salaries and how cost containment influence their work.

### Strengths and limitations

A major strength of this study is that, because of the size of Iceland’s population, we were able to invite almost all (96%) of the doctors in the country to participate in it; the response rate of 61% is relatively high for doctors. Although this represents only 55% of all specialists in the country, all specialities are well represented. A potential weakness of the study is that all measures are self-reported. Furthermore, because this is a cross-sectional study, we cannot make inferences about the causal effects of the statistically significant predictor variables.

## Conclusions

Two-thirds of Iceland’s specialist doctors have considered moving abroad. Cost-containment initiatives at work and worries about personal finances led them to consider moving abroad, given the influence of other factors. In particular, the fact that the youngest specialists, most of whom have only recently returned from overseas, are considering working abroad, should demand attention.

## Competing interests

All authors declare that there was no support from any organization for the submitted work; no financial relationships with any organizations that might have an interest in the submitted work; no other relationships or activities that could appear to have influenced the submitted work.

## Authors’ contributions

IBS, RT, and KT designed the study. IBS collected the data. IBS and RT had full access to all the data and did the main statistical analysis. All authors contributed to the interpretation of the data. IBS and RT drafted the manuscript and KT and OA participated in writing the manuscript. All authors read and approved the final manuscript.

## Pre-publication history

The pre-publication history for this paper can be accessed here:

http://www.biomedcentral.com/1472-6963/13/524/prepub

## References

[B1] Pang T Lansang MA Haines A Brain drain and health professionals BMJ 2002 13 499 500 10.1136/bmj.324.7336.499 11872536 PMC1122434

[B2] Karanikolos M Mladovsky P Cylus J Thomson S Basu S Stuckler D Mackenbach JP Mckee M Financial crisis, austerity, and health in Europe Lancet 2013 13 1323 31 10.1016/S0140-6736(13)60102-6 23541059

[B3] Olafsdottir H Current concerns in Icelandic psychiatry; nation in crisis Nord J Psychiatry 2009 13 188 89 10.1080/08039480902826850 19280382

[B4] Bjornsdottir K Nelson S Health and vulnerability in a time of economic uncertainty Nurs Inq 2010 13 1 10.1111/j.1440-1800.2010.00485.x 20137025

[B5] Jónsson S Health care system at a crossroads Icelandic Med J 2011 13 211 editorial. *Læknablaðið* 10.17992/lbl.2011.04.359 21451198

[B6] UNDP Human development report 2007/2008 http://hdr.undp.org/en/media/HDR_20072008_EN_Indicator_tables.pdf

[B7] Mackenbach JP McKee M A comparative analysis of health policy performance in 43 European countries Eur J Public Health 2013 13 195 201 10.1093/eurpub/cks192 23402806

[B8] Statistics Icelandhttp://www.statice.is/

[B9] Thomson S Osborn R Squires D Jun M International profiles of health care systems, 2012 2012 The Commonwealth fund http://www.commonwealthfund.org/~/media/Files/Publications/Fund%20Report/2012/Nov/1645_Squires_intl_profiles_hlt_care_systems_2012.pdf

[B10] The budget for 2008, ‘Fjárlögin 2008’ http://www.althingi.is/altext/135/s/pdf/0507.pdf

[B11] The budget for 2010, ‘Fjárlögin 2010’ http://www.althingi.is/altext/138/s/pdf/0594.pdf

[B12] Frjáls verslunjournal in paper edition only): August 2009 (the salaries in 2008 of about 200 doctors) and August 2010 (the salaries in 2009 of the same doctors). We excluded 8 doctors who had over 100% reduction or increase in their salaries 2009 Reykjavík: Heimur hf

[B13] Statistics Iceland http://www.statice.is/Pages/444?NewsID=8972

[B14] Vanasse A Scott S Courteau J Orzanco MG Canadian family physicians’ intention to migrate: associated factors Can Fam Physician 2009 13 396 397 19366952 PMC2669015

[B15] Landon BE Reschovsky JD Pham HH Blumenthal D Leaving medicine, the consequences of physician dissatisfaction Med Care 2006 13 234 242 10.1097/01.mlr.0000199848.17133.9b 16501394

[B16] Commuting: Iceland’s challenge and opportunity Nordic Labour Journal 2012 http://www.nordiclabourjournal.org/i-fokus/in-focus-2012/when-commuting-becomes-an-obstacle-race/article.2012-04-13.6268727380

[B17] Aasland OG Rosta J Nylenna M Healthcare reforms and job satisfaction among doctors in Norway Scand J Public Health 2010 13 253 258 10.1177/1403494810364559 20215483

[B18] Solberg IB Ro KI Aasland O Gude T Moum T Vaglum P Tyssen R The impact of change in a doctor’s job position: a five-year cohort study of job satisfaction among Norwegian doctors BMC Health Serv Res 2012 13 41 10.1186/1472-6963-12-41 22340521 PMC3342917

[B19] Warr P Cook J Wall T Scales for the measurement of some work attitudes and aspects of psychological well-being J Occup Psychol 1979 13 129 148 10.1111/j.2044-8325.1979.tb00448.x

[B20] Rosta J Nylenna M Aasland OG Job satisfaction among hospital doctors in Norway and Germany. A comparative study on national samples Scand J Public Health 2009 13 503 508 10.1177/1403494809106504 19535404

[B21] Mathers N Jones N Hannay D Heartsink patients: a study of their general practitioners Br J Gen Pract 1995 13 293 296 7619583 PMC1239262

[B22] Manski CF The use of intentions data to predict behavior: a best-case analysis J Am Stat Assoc 1990 13 934 40 10.1080/01621459.1990.10474964

[B23] Bertrand M Mullainathan S Do people mean what they say? Implications for subjective survey data Am econ rev 2001 13 67 72 10.1257/aer.91.2.67

[B24] Gordon I Molho I Duration dependence in migration behaviour: cumulative inertia versus stochastic change Environ and plann 1995 13 1961 75 10.1068/a271961 12291218

[B25] Böheim R Taylor M Tied down or room to move? Investigating the relationships between housing tenure, employment status and residential mobility in britain Scott j of pol econ 2002 13 369 92 10.1111/1467-9485.00237

[B26] Mullan F The metrics of the physician brain drain N Engl J Med 2005 13 1810 8 10.1056/NEJMsa050004 16251537

[B27] Shortage in MDs Looming 2010 Iceland review online http://www.icelandreview.com/icelandreview/daily_news/Shortage_in_MDs_Looming_0_363996.news.aspx

[B28] Imminent collapse in Iceland’s Healthcare System 2011 Iceland review online http://www.icelandreview.com/icelandreview/search/news/Default.asp?ew_0_a_id=380301

[B29] Oberoi SS Lin V Brain drain of doctors from southern Africa: brain gain for Australia Aust Health Rev 2006 13 25 33 16448375

[B30] Astor A Akhtar T Matallana MA Muthuswamy V Olowu FA Tallo V Lie RK Physician migration: views from professionals in Colombia, Nigeria, India, Pakistan and the Philippines Soc Sci Med 2005 13 2492 500 10.1016/j.socscimed.2005.05.003 15953667

[B31] García-Pérez MA Amaya C Otero Á Physicians’ migration in Europe: an overview of the current situation BMC Health Serv Res 2007 13 201 10.1186/1472-6963-7-201 18070353 PMC2248190

[B32] Costigliola V Mobility of medical doctors in cross-border healthcare (review article) EPMA J 2011 13 333 339 10.1007/s13167-011-0133-7 23199171 PMC3405407

[B33] Williams AM Baláz V International return mobility, learning and knowledge transfer: a case study of Slovak doctors Soc Sci Med 2008 13 1924 33 10.1016/j.socscimed.2008.09.003 18845371

[B34] Holt E Czech doctors resign en masse Lancet 2011 13 111 112 10.1016/S0140-6736(11)60004-4 21226228

[B35] Dussault G Fronteira I Cabral J Migration of health personnel in the WHO European Region 2009 WHO http://www.euro.who.int/__data/assets/pdf_file/0010/95689/E93039.pdf

[B36] Kopetsch T The migration of doctors to and from Germany J Public Health 2008 13 1924 33

[B37] Probst TM Barling J, Kelloway EK, Frone MR Handbook of work stress Economic stressors 2005 Thousand Oaks, CA: Sage 267 297

[B38] Shoss MK Probst TM Perrewé PL, Halbesleben JRB, Rosen CC Multilevel outcomes of economic stress: an agenda for future research The role of the Economic Crisis on Occupational Stress and Well Being. Research in Occupational Stress and Well Being, Volume 10 2012 Bingley, UK: Emerald Group Publishing Limited 43 86

[B39] Mejia A Migration of physicians and nurses: a world wide picture. 1978 Bull World Health Organ 2004 13 626 630 15457641 PMC2622933

[B40] Brown RP Connell J The migration of doctors and nurses from South Pacific Island Nations Soc Sci Med 2004 13 2193 210 10.1016/j.socscimed.2003.08.020 15047077

[B41] Benarroch M Grant H The interprovincial migration of Canadian physicians: does income matter? Appl Econ 2004 13 2335 45 10.1080/0003684042000281543

[B42] Spickard A Jr Gabbe SG Christensen JF Mid-career burnout in generalist and specialist physicians JAMA 2002 13 1447 50 10.1001/jama.288.12.1447 12243624

[B43] Gravelle H National survey of job satisfaction and retirement intentions among general practitioners in England BMJ 2003 13 22 10.1136/bmj.326.7379.22 12511457 PMC139500

[B44] Wallace JE Lemaire JB Ghali WA Physician wellness: a missing quality indicator Lancet 2009 13 1714 21 10.1016/S0140-6736(09)61424-0 19914516

[B45] Basu K Rajbhandary S Interprovincial migration of physicians in Canada: what are the determinants? Health Policy 2006 13 186 193 10.1016/j.healthpol.2005.06.003 16026890

[B46] Stilwell B Diallo K Zurn P Dal Poz MR Adams O Buchan J Developing evidence-based ethical policies on the migration of health workers: conceptual and practical challenges Hum Resour Health 2003 13 8 10.1186/1478-4491-1-8 14613524 PMC272935

[B47] Williams ES Understanding physicians’ intentions to withdraw from practice: the role of job satisfaction, job stress, mental and physical health Health Care Manage rev 2001 13 7 19 11233355 10.1097/00004010-200101000-00002

[B48] Stilwell B Diallo K Zurn P Vujicic M Adams O Adams O Dal Poz M Migration of health-care workers from developing countries: strategic approaches to its management Bull World Health Organ 2004 13 595 600 15375449 PMC2622931

